# The effects of eight weeks of sand-based plyometric training on lower-extremity explosive strength, balance, and agility in male collegiate badminton players

**DOI:** 10.3389/fphys.2025.1708619

**Published:** 2025-11-26

**Authors:** Nijiao Deng, Xin Zheng, Hairong Wang, Ziren Zhao, Xu Xia, Hangshuo Zhang, Kaixiang Zhou, Bin Zhang

**Affiliations:** 1 College of Physical Education and Health Science, Chongqing Normal University, Chongqing, China; 2 College of Physical Education and Health Science, Zhejiang Normal University, Jinhua, China

**Keywords:** plyometric training, lower-extremity explosive strength, dynamic balance, agility, badminton players

## Abstract

**Background:**

Badminton is a high-intensity sport that demands explosive strength, dynamic balance, and agility. Plyometric training (PT) is crucial for enhancing these abilities, yet there is limited research comparing sand-based PT to hard-surface PT in badminton players.

**Objective:**

This study compared the effects of sand-based PT and hard-surface PT on lower-extremity explosive strength, dynamic balance, and agility in male badminton players.

**Methods:**

Twenty-eight male collegiate badminton players were randomly assigned to a sand-based PT group (n = 14) or a hard-surface PT group (n = 14). Both groups performed plyometric programs twice weekly for 8 weeks. Outcomes measurement before and after training included countermovement jump (CMJ) height, squat jump (SJ) height, drop jump (DJ) height, standing long jump (SLJ) distance, Y-balance test (YBT), hexagon jump test, and badminton-specific agility test (lateral agility test and four-corner agility test). We used two-way repeated measures analysis of variance (ANOVA) (time × group) with Bonferroni *post hoc* tests, and effect sizes were reported as partial eta squared ( $\eta_{p}^{2}$ ) or Cohen’s d (p < 0.05).

**Results:**

Statistical analysis revealed significant time main effects across multiple metrics: CMJ height (F = 81.26, p < 0.01, $\eta_{p}^{2}$ = 0.75), SJ height (F = 82.86, p < 0.01, $\eta_{p}^{2}$ = 0.76), DJ height (F = 54.58, p < 0.01, $\eta_{p}^{2}$ = 0.68, large effect), SLJ distance (F = 49.86, p < 0.01, $\eta_{p}^{2}$ = 0.66), dynamic balance ability (p < 0.01), and agility (p < 0.01), no significant between-group differences were found for any of the variables (p > 0.05). Additionally, a significant interaction effect between time and group was observed in the left leg to right (F = 4.76, p = 0.04).

**Conclusion:**

This study indicates that both sand-based PT and hard-surface PT over 8 weeks significantly enhance explosive power, dynamic balance, and agility performance, with no significant differences between groups.

## 1 Introduction

Explosive strength, dynamic balance, and agility are critical performance components in badminton, a sport characterized by rapid changes in direction, dynamic lunges, and powerful lower-extremity actions (Manrique and González-Badillo, 2003; Phomsoupha and Laffaye, 2015). Enhancing these physical attributes is crucial for badminton players aiming to improve competitive performance and reduce injury risk. Plyometric training (PT) has long been recognized as an effective method for enhancing athletic performance, such as jump height, reactive strength, and agility (Asadi et al., 2016; Bouguezzi et al., 2020; Fischetti et al., 2018; Ziv and Lidor, 2010). For example,. A meta-analysis specific to badminton indicates that PT yields small to moderate improvements in performance outcomes, including strength, agility, speed, and balance (Deng et al., 2024). Consistent with this evidence, a systematic review concludes that PT also benefits agility, strength, speed, and jump performance (Shedge et al., 2024). Alikhani et al. (Alikhani et al., 2019) demonstrated that a 6-week PT program significantly enhanced dynamic balance and knee proprioception in female badminton players. Bozdogan et al. (Bozdoğan and Kızılet, 2017) reported notable improvements in agility and jump performance following an 8-week PT in male badminton players. Similarly, Chandra et al. (Chandra et al., 2023) observed that a 3-week PT program led to significant gains in agility, sprint speed, and explosive strength among male collegiate badminton players. The key mechanism for improving athletic performance on PT lies mainly in the stretch-shortening cycle (SSC) of muscle fibers, whereby the elastic energy stored during the eccentric contraction phase is converted into mechanical energy during the concentric contraction phase (Heinecke, 2021; Wilk et al., 1993). Additionally, proprioceptive feedback from muscle stretching sends signals to the spinal cord, activating α-motor neurons (Davies et al., 2015). This process enhances agonist muscle activation, recruits motor units, and inhibits antagonist muscle activity (Deng et al., 2023). Although multiple studies have demonstrated that PT enhances badminton players performance (Ozmen and Aydogmus, 2017; Panda et al., 2022), existing training protocols are predominantly implemented on hard-surfaces. Recent studies have suggested that performing plyometric training on unstable or softer surfaces, such as sand, may offer unique training benefits (Arazi et al., 2016; Hammami et al., 2020; Pereira et al., 2023a). Using a sand surface for PT may enhance neuromuscular performance (Ahmadi et al., 2021; Pereira et al., 2021). Due to its unstable and compliant nature, sand can increase muscle activation (Pereira et al., 2021). Moreover, sand’s absorptive properties attenuate impact forces, thereby reducing tissue and joint stress while increasing metabolic demand compared with harder surfaces (Impellizzeri et al., 2008). It is essential to investigate the effects of PT on different surfaces when implementing PT in sports training practices. Research on the effects of PT under unstable ground conditions in badminton remains largely unexplored. Guo et al. conducted an 8-week program that combined balance training with PT, resulting in improved change-of-direction (COD) performance and reduced lower-limb injury risk in young badminton athletes (Guo and Xu, 2025). Likewise, Zhang et al. found that a 6-week balance and PT intervention significantly decreased time to stabilization (TTS) after single-leg landings and enhanced postural control, as indicated by center-of-pressure (COP) metrics (Zhang et al., 2024). It is worth noting that these studies used a combined approach of balance training (BOSU balls, Swiss balls, and balance pads) with physical therapy, rather than isolating “ground type.” Therefore, their conclusions about ground properties are limited. Furthermore, there is an ongoing debate in the literature about the impact of surface type on PT. For instance, Impellizzeri et al. reported that participants in the unstable surface (such as grass) PT group exhibited significantly greater improvements in countermovement jump (CMJ) height compared to hard-surface PT (23); but Lucas A. Pereira and colleagues observed no significant improvement in vertical jump performance following an 8-week unstable surface (such as sand) PT in elite youth soccer players (Pereira et al., 2023b). The inconsistency in the results of the above studies may stem from differences in participant characteristics, training protocols. Given the current absence of empirical evidence on the effects of sand-based PT in badminton players. It is unclear whether this specific PT protocol is also effective in enhancing explosive strength, balance, and agility in badminton players.

Therefore, this study aims to investigate the effects of an 8-week sand-based PT program on lower-extremity explosive strength, balance, and agility in male collegiate badminton players, providing insights into its potential as an effective training strategy in badminton-specific conditioning. We hypothesized that sand-based PT enhances lower extremity explosive strength and agility similarly to hard-surface PT; however, sand-based PT offers superior improvements in dynamic balance compared to hard-surface PT.

## 2 Materials and methods

### 2.1 Participants

An *a priori* power analysis (G*Power 3.1.9.7) with a large expected effect size (f = 0.57, from Mehrez Hammami) (Hammami et al., 2020), α = 0.05, and power = 0.80 indicated that 27 participants (13–14 per group) were required.

**FIGURE 1:**
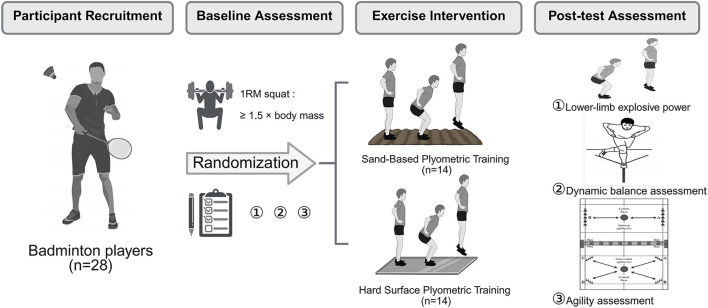
Flow chart of the experiment.

Twenty-eight male collegiate badminton players (age:21.0 ± 1.73 years; body mass: 68.32 ± 12.72 kg; height: 174.85 ± 6.10 cm) participated in this study. Participants were randomly assigned to either the sand-based PT group (n = 14) or the hard-surface PT group (n = 14). All participants were healthy, non-smokers, and not taking any medications or supplements; participants had a 1RM barbell squat weight ≥1.5 times their body weight; had no history of lower extremity injury in the last 3 months; and were all right-handed racket users. The study was approved by the Ethics Committee of the College of Physical Education and Health Science Chongqing Normal University (No. NCNU-PSY-202508-013), and all procedures were in accordance with the Declaration of Helsinki. Before the experiment, participants were informed of the benefits and potential risks related to the study, and all signed the informed consent form (Figure 1).

### 2.2 Study design

This study used an 8-week, single-blind (assessor-blind) randomized controlled trial (Figure 1). Using a computer-generated randomization sequence, participants were randomly assigned to either the sand-based PT group (n = 14) or the hard-surface PT group (n = 14). Both groups followed the training protocol twice weekly, with a 24–48-h recovery period between sessions. Participants were prohibited from engaging in high-intensity training outside of the experimental protocol during the intervention period to control for potential confounding variables. All participants completed outcome measurements before (baseline) and after the intervention. Participants were familiarized with the testing procedures and training program 1 week before baseline testing. At the same time, under the guidance of certified strength and conditioning coaches, they completed a barbell half squat one-rep maximum (1RM) test according to NSCA guidelines (Pearson et al., 2000). Demographic information and anthropometric data (weight and height) were recorded. All evaluation sessions were conducted on the same pitch and at the same time of day for each participant to prevent any potential confounding effects related to circadian rhythms. Temperature (21.2 °C ± 0.3 °C) and humidity (29.0% ± 0.4%) were maintained consistently. Participants were instructed to avoid vigorous exercise for 48 h before each evaluation day and to refrain from consuming alcohol and caffeine for 24 h before the sessions.

#### 2.2.1 Training protocol

The sand-based PT group trained on a standard beach volleyball court, while the hard-surface PT group trained on a standard athletics track. The training protocol followed the “progressive overload” principle outlined by Ramirez-Campillo et al. (2020). It was structured into three phases: an adaptation phase (weeks 1–2), an intensive enhancement phase (weeks 3–5), and a maintenance phase (weeks 6–8). Both groups of participants completed the same number of jump repetitions, with ground contacts per training session ranging from 150 to 200. Each session included five to six plyometric exercises (see Supplementary Material Table 1). Training sessions lasted approximately 50 min and began with a warm-up consisting of 5 min of jogging followed by six dynamic stretching drills. Participants were encouraged to exert maximum effort during all training sessions, with repetitions separated by recovery intervals of 15–30 s and group rest periods of 3 min.

#### 2.2.2 Outcomes measurement

##### 2.2.2.1 Explosive strength

We utilized CMJ height, squat jumps (SJ) height, drop jumps (DJ) height, and standing long jumps (SLJ) distance to evaluate explosive strength in participants. Each test was conducted three times, and the highest value from the trials was recorded for analysis. Participants completed CMJ height, SJ height, and DJ height tests on a force platform (Kistler Group, Switzerland) with a sampling frequency of 1,000 Hz. The SLJ distance test was conducted indoors on a wooden floor.

##### 2.2.2.2 Dynamic balance

We used the Y-balance test to assess participants’ dynamic balance. Participants stood on one leg and reached as far as possible in three directions (anterior, posteromedial, and posterolateral) with the contralateral leg. Adhesive tape marked the floor directions with 5 mm increments for precise measurement. Each participant was allowed a maximum of three attempts in each direction, and a short rest break (10–15 s) was provided to reduce fatigue. The maximum distance (to the nearest 0.5 cm) over the three trials was recorded and analyzed. The participant’s lower limb reach was normalized to limb length. The limb length was measured from the anterior superior iliac spine to the most distal portion of the medial malleolus. The normalized value was calculated as the reach distance divided by the limb length and then multiplied by 100%, which expressed the reach distance as a percentage of the limb length. Total reach distance was the sum of the three successful reach directions divided by three times limb length, and at the end, multiplied by 100% (Alikhani et al., 2019) (Supplementary Material Figure 1).

##### 2.2.2.3 Hexagon agility

We utilized the hexagon jump test to assess the agility of the participants. The participants stood 50 cm behind the No. 1 side of the hexagon, and after hearing the command of “Ready, go”, they quickly completed jumping in and out of the line with a circle clockwise from 1 to 6. Timing was conducted with a handheld stopwatch, recording the time from the start command to the completion of the circuit. A total of three trials were performed, and the shortest time was recorded for analysis. There was a 2-min passive rest between the two tests (Lu et al., 2022) (Supplementary Material Figure 2).

##### 2.2.2.4 Badminton-specific agility

Badminton-specific agility test was modified based on Hughes’ badminton-specific ability test (Hughes and Bopf, 2005). This test includes lateral (lateral agility test) and diagonal mobility with abrupt changes in direction (four-corner agility test) (Supplementary Material Figure 3). Participants position themselves facing the net at the center of the court, starting the test in the ready position for a badminton match. The lateral agility test required participants to shuffle laterally across the width of the court for a total of 10 repetitions, striking each of the upturned shuttlecocks placed on the line marking the outside of the singles court. The four-corner agility test required participants to move around the four corners of the court for a total of 16 repetitions in a specific sequence of four directions, striking each upturned shuttlecock placed at each corner. Testers used stopwatches to record the movement times of the participants. A recovery period of ≥10 min is required between lateral and diagonal tests to ensure the participants’ condition is optimal (Hughes and Bopf, 2005).

### 2.3 Statistical analysis

Data were presented as Mean ± SD. The normality of data was tested using the Shapiro–Wilk test. The effect of significance on outcomes was analyzed using a two-way (time × group) repeated measures analysis of variance, and with *post hoc* analyses done with the Bonferroni test. Repeated-measurement data needed to satisfy Mauchly’s test (p > 0.05); When Mauchly’s test was violated, the Greenhouse-Geisser correction was applied. If no interaction between time and group was observed, the data were analyzed for the main effect, and then effect sizes (partial eta-squared [ $\eta_{p}^{2}$ ]) were calculated, where effect sizes were categorized as trivial ( $\eta_{p}^{2}$ <0.01), small (0.01≤ $\eta_{p}^{2}$ <0.06), moderate (0.06≤ $\eta_{p}^{2}$ <0.14), and large ( $\eta_{p}^{2}$ >0.14) effects. In contrast, if an interaction between time and group was detected, the data were analyzed for simple effects, and effect sizes were calculated (Cohen’s d), with <0.5, 0.5 ≤ x ≤ 0.8, and >0.8 considered small, moderate, and large, respectively (Cohen, 2013). p < 0.05 was considered statistically significant. All analyses were performed using the SPSS statistical package (version 25.0, IBM Statistics, Chicago, IL).

## 3 Results

### 3.1 Explosive strength

#### 3.1.1 CMJ height

**FIGURE 2:**
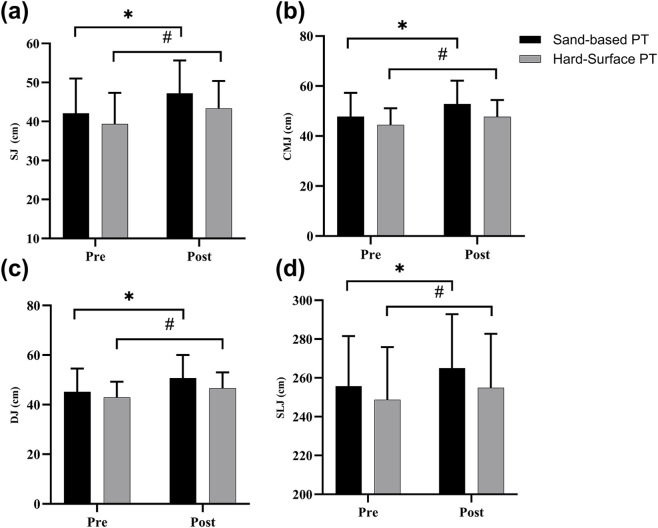
The effects of plyometric training on lower-extremity explosive strength. Values are presented as mean ± SD. **(a)** Squat jump (SJ); **(b)** countermovement jump (CMJ); **(c)** drop jump (DJ); **(d)** standing long jump (SLJ). ^*^ indicates a significant difference from baseline in the Sand-based PT group; ^#^ indicates a significant difference from baseline in the Hard-surface PT group.

CMJ height reliability across three measurements: ICC = 0.996. A non-significant interaction effect between time and group was observed (F = 4.09, p = 0.053, $\eta_{p}^{2}$ = 0.14, large effect). The time main effects analysis showed that both the Sand-based PT and Hard-surface PT exhibited a significant increase in CMJ height compared to baseline (F = 81.26, p < 0.01, $\eta_{p}^{2}$ = 0.75, large effect). The group main effects analysis showed that there was no significant difference in CMJ height between the Sand-based PT and Hard-surface PT groups (F = 1.93, p = 0.18, $\eta_{p}^{2}$ = 0.07, moderate effect) (Figure 2a) (Supplementary Material Tables 2).

#### 3.1.2 SJ height

SJ height reliability across three measurements: ICC = 0.997. A non-significant interaction effect between time and group was observed (F = 1.22, p = 0.28, $\eta_{p}^{2}$ = 0.05 small effect). The time main effects analysis showed that both the Sand-based PT and Hard-surface PT exhibited a significant increase in SJ height compared to baseline (F = 82.86, p < 0.01, $\eta_{p}^{2}$ = 0.76, large effect). The group main effects analysis showed that there was no significant difference in SJ height between the Sand-based PT and Hard-surface PT groups (F = 1.93, p = 0.29, $\eta_{p}^{2}$ = 0.04, small effect) (Figure 2b).

#### 3.1.3 DJ height

DJ height reliability across three measurements: ICC = 0.997. A non-significant interaction effect between time and group was observed (F = 1.93, p = 0.18, $\eta_{p}^{2}$ = 0.07, moderate effect). The time main effects analysis showed that both the Sand-based PT and Hard-surface PT groups exhibited a significant increase in DJ height compared to baseline (F = 54.58, p < 0.01, $\eta_{p}^{2}$ = 0.68, large effect). The group main effects analysis showed that there was no significant difference in DJ height between the Sand-based PT and Hard-surface PT groups (F = 1.14, p = 0.29, $\eta_{p}^{2}$ = 0.04, small effect) (Figure 2c).

##### 3.1.3.1 SLJ distance

SLJ distance reliability across three measurements: ICC = 0.995. A non-significant interaction effect between time and group was observed (F = 2.14, p = 0.16, $\eta_{p}^{2}$ = 0.08, moderate effect). The time main effects analysis showed that both the Sand-based PT and Hard-surface PT groups exhibited a significant increase in SLJ distance compared to baseline (F = 49.86, p < 0.01, $\eta_{p}^{2}$ = 0.66, large effect). The group main effects analysis showed that there was no significant difference in SLJ distance between the Sand-based PT and Hard-surface PT groups (F = 0.71, p = 0.41, $\eta_{p}^{2}$ = 0.03, small effect) (Figure 2d).

### 3.2 Dynamic balance

#### 3.2.1 Non-dominant (left leg)

Non-dominant leg reliability across three measurements: ICC = 0.964 to 0.986. A non-significant interaction effect between time and group was observed in the left leg to forward (F = 0.71, p = 0.41, $\eta_{p}^{2}$ = 0.03, small effect) and left leg to left (F = 1.09, p = 0.31, $\eta_{p}^{2}$ = 0.04, small effect) direction. The time main effects analysis showed that both the Sand-based PT and Hard-surface PT groups exhibited a significant increase in left leg to forward (F = 50.37, p < 0.01, $\eta_{p}^{2}$ = 0.66, large effect) and left leg to left (F = 89.70, p < 0.01, $\eta_{p}^{2}$ = 0.78, large effect) compared to baseline. The group main effects analysis showed that there was no significant difference in left leg to forward (F = 0.29, p = 0.59, $\eta_{p}^{2}$ = 0.01, small effect) and left leg to left (F = 0.86, p = 0.36, $\eta_{p}^{2}$ = 0.03, small effect) between the Sand-based PT and Hard-surface PT groups.

**FIGURE 3:**
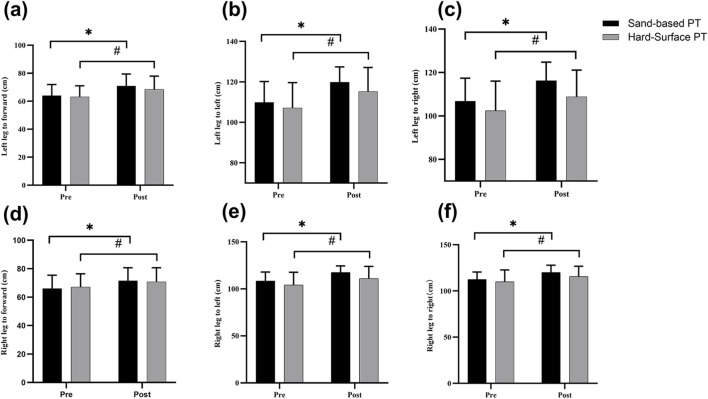
The effects of plyometric training on dynamic balance assessment. Values are presented as mean ± SD. **(a)** Left leg to forward; **(b)** left leg to left; **(c)** left leg to right; **(d)** right leg to forward; **(e)** right leg to left; **(f)** right leg to right. ^*^ indicates a significant difference from baseline in the Sand-based PT group; ^#^ indicates a significant difference from baseline in the Hard-surface PT group.

A significant interaction effect between time and group was observed in the left leg to right (F = 4.76, p = 0.04). The time main effects analysis revealed that the Sand-based PT group showed statistically significant improvements from baseline (p < 0.01, d = 0.99, large effect), and the Hard-surface PT group demonstrated statistically significant improvements from baseline (p < 0.01, d = 0.50, moderate effect); The group main effects revealed no significant difference (p = 0.08, d = 0.71, moderate effect) (Figure 3).

#### 3.2.2 Dominant leg (right leg)

Dominant leg reliability across three measurements: ICC = 0.969 to 0.976. A non-significant interaction effect between time and group was observed in the right leg to forward (F = 3.73, p = 0.06, $\eta_{p}^{2}$ = 0.17, large effect), right leg to right (F = 2.05, p = 0.16, $\eta_{p}^{2}$ = 0.07, moderate effect), and right leg to left (F = 2.16, p = 0.15, $\eta_{p}^{2}$ = 0.07, moderate effect) direction. The time main effects analysis showed that both the Sand-based PT and Hard-surface PT groups exhibited significant increase in the right leg to forward (F = 90.44, p < 0.01, $\eta_{p}^{2}$ = 0.77, large effect), the right leg to right (F = 93.20, p < 0.01, $\eta_{p}^{2}$ = 0.78, large effect) and right leg to left (F = 113.67, p < 0.01, $\eta_{p}^{2}$ = 0.81, large effect) compared to baseline. The group main effects analysis showed that there was no significant difference in right leg to forward (F = 0.002, p = 0.97, $\eta_{p}^{2}$ = 0.001, trivial effect), the right leg to right (F = 0.88, p = 0.36, $\eta_{p}^{2}$ = 0.03, small effect) and right leg to left (F = 1.73, p = 0.20, $\eta_{p}^{2}$ = 0.06, moderate effect) between the Sand-based PT and Hard-surface PT groups (Figure 3).

### 3.3 Agility

#### 3.3.1 Hexagon jump test

**FIGURE 4:**
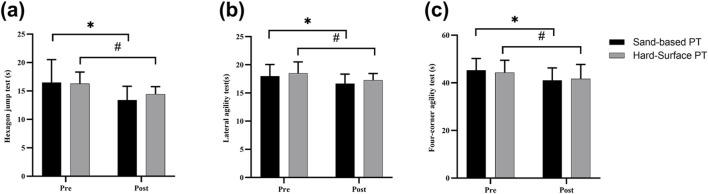
The effects of plyometric training on agility assessment. Values are presented as mean ± SD. **(a)** Hexagon jump test; **(b)** lateral agility test; **(c)** four-corner agility test. ^*^ indicates a significant difference from baseline in the Sand-based PT group; ^#^ indicates a significant difference from baseline in the Hard-surface PT group.

Hexagon jump test reliability across three measurements: ICC = 0.833. A non-significant interaction effect between time and group was observed (F = 1.52, p = 0.23, $\eta_{p}^{2}$ = 0.06, moderate effect). The time main effects analysis showed that both the Sand-based PT and Hard-surface PT groups showed significant improvement (reduced time) in the hexagon jump compared to baseline (F = 23.54, p < 0.01, $\eta_{p}^{2}$ = 0.48, large effect). The group main effects analysis showed that there was no significant difference in the hexagon jump test between the Sand-based PT and Hard-surface PT groups (F = 0.25, p = 0.63, $\eta_{p}^{2}$ = 0.009, trivial effect) (Figure 4A).

#### 3.3.2 Badminton-specific agility

##### 3.3.2.1 Lateral agility test

A non-significant interaction effect between time and group was observed (F = 0.03, p = 0.86, $\eta_{p}^{2}$ = 0.001 trivial effect). The time main effects analysis showed that both the Sand-based PT and Hard-surface PT groups showed significant improvement (reduced time) in the lateral agility test compared to baseline (F = 26.48, p < 0.01, $\eta_{p}^{2}$ = 0.51, large effect). The group main effects analysis showed that there was no significant difference in the lateral agility test between the Sand-based PT and Hard-surface PT groups (F = 0.79, p = 0.38, $\eta_{p}^{2}$ = 0.03, small effect) (Figure 4b).

##### 3.3.2.2 Four-corner agility test

A non-significant interaction effect between time and group was observed (F = 1.54, p = 0.23, $\eta_{p}^{2}$ = 0.06, moderate effect). The time main effects analysis showed that both the Sand-based PT and Hard-surface PT groups showed significant improvement (reduced time) in the four-corner agility test compared to baseline (F = 31.89, p < 0.01, $\eta_{p}^{2}$ = 0.55, large effect). The group main effects analysis showed that there was no significant difference in the Four-Corner agility test between the Sand-based PT and Hard-surface PT groups (F = 0.008, p = 0.93, $\eta_{p}^{2}$ = 0.001, trivial effect) (Figure 4c).

## 4 Discussion

This study investigated the effects of an 8-week sand and hard-surface PT on lower-extremity explosive strength, dynamic balance, and agility performance in collegiate badminton players. The results showed that both sand-based PT and hard-surface PT groups achieved significant improvements in lower-extremity explosive strength, dynamic balance and agility, with no significant differences between groups.

Following PT, lower-body explosive power similarly increased, regardless of training surface. Our findings align with a meta-analysis: improvements in jumping ability are comparable regardless of whether PT is conducted on sandy or hard-surfaces (Pereira et al., 2021). Although sand surfaces increase shock absorption and compromise force application during explosive actions (Giatsis et al., 2004; Giatsis et al., 2018), this may hamper explosive strength. We confirmed that sand-based PT effectively increases jump power. For example, Mina Ahmadi et al. discovered that 8 weeks of sand-based PT was effective in enhancing the vertical jump of female volleyball players (Ahmadi et al., 2021). Likewise, a 7-week sand-based PT intervention induced large and significant improvements in vertical jump performance among junior male handball players (Hammami et al., 2020). Based on this, we hypothesize that adaptations from sand-based PT, substantial elastic energy is dissipated, increasing energy expenditure and muscle activation levels, thereby enhancing motor-unit recruitment during explosive actions (e.g., CMJ height, SJ height, DJ height, SLJ distance). These hypotheses are supported by prior research: muscle activation and jumping ability significantly increase after sand-based PT (Häkkukinen et al., 1985; Mirzaei et al., 2014). However, this remains speculative, and further studies are necessary to explore and clarify these mechanisms. Conversely, adaptations during hard-surface PT may be to improved efficiency in storing and reusing elastic energy during explosive actions (Behm and Sale, 1985). Thus, sand and hard-surface PT enhance jump performance through distinct, potentially complementary mechanisms.

Contrary to this study’s assumptions, the differences between sand-based and hard-surface PT in balance outcomes were not statistically significant. Previous studies have demonstrated that combined PT and balance training yields greater benefits than PT alone in enhancing balance-related abilities (Guo and Xu, 2025; Chaouachi et al., 2014). Guo et al. found that an 8-week, 3-times-per-week combined PT and balance training significantly improved dynamic balance, neuromuscular control, and postural stability in young male badminton athletes compared with PT alone (Guo and Xu, 2025). Chaouachi et al. reported that, in healthy boys aged 12–15, combined PT and balance training produced greater effect sizes in balance outcomes than PT alone (Chaouachi et al., 2014). Given the findings of Guo et al. and Chaouachi et al., this study regarding balance ability was quite unexpected. We speculate that the underlying cause may lie in differences in training content: For example, in addition to training on unstable balance pads, Guo et al.'s training program incorporated balance exercises using equipment such as BOSU balls and Swiss balls (Guo and Xu, 2025). Similarly, Chaouachi et al.'s training program includes one specialized balance exercise (i.e., single-legged squats on a hemispherical dome) in addition to four PT during each training session (Chaouachi et al., 2014). Therefore, the “significant differences” reported in previous studies are more likely attributable to additional balance training rather than solely due to surface instability during training. Future research aiming to improve balance should consider incorporating specialized balance exercises into PT.

This study found that both sand-based PT and hard-surface PT groups effectively improved badminton players’ agility, but no significant differences were observed between the groups. This outcome is contrary to that of Guo et al. (2021), who found that balance training (i.e., BOSU ball, Swiss ball, and Balance pad) combined with hard-surface PT can effectively improve the agility of badminton players (Guo et al., 2021). It is difficult to explain this result, but it might be related to agility tests, such as the Hexagon jump test and badminton-specific agility tests, were conducted on hard-surfaces. These surfaces differ significantly from training stimuli, like sand, which dilutes the transfer effect. In addition, this study used total time metrics, which may have obscured subtle improvements in movement quality (such as center of gravity control and start-stop changes), advantages that are difficult to highlight in time tests. Several studies have compared stable and unstable training surfaces, consistently showing that athletes can achieve comparable gains in agility, regardless of whether they train on sand, hard-surfaces, or grass (Pereira et al., 2023a; Pereira et al., 2023b; Ramirez-Campillo et al., 2020; Yu et al., 2025; Modena et al., 2025). It is worth noting that if the training intensity or frequency is too high, the “additional stability load” of sand may be converted into fatigue rather than improved agility. We hypothesize that sand-based PT improves lower limb stability, but hard-surface training is more effective in improving badminton-specific agility. Future research could investigate the differences between sand-based PT, hard-surface PT, and a combined sand and hard-surface PT protocol for enhancing badminton-specific agility.

Although some results did not reach statistical significance, the small-to-moderate effect sizes in vertical jump and balance are important. Lower-body explosiveness and balance are crucial for badminton movements like acceleration and jump smashes, helping athletes maintain stability (Ghorpade et al., 2024; Lu et al., 2025). Even slight improvements can enhance performance in elite competition, which may become more evident with longer interventions or larger samples.

### 4.1 Limitation

Several limitations of this pilot study should be noted. First, results are limited to male collegiate athletes, and future studies should include female or youth players to improve generalizability. Second, all post-intervention tests were conducted on a hard-surface, which might have slightly favored the hard-surface group in performance outcomes. Third, no physiological or neuromuscular measures (e.g., EMG, GRF) were included, which restricts insights into the underlying mechanisms of adaptation. Finally, this study did not collect detailed vertical-jump kinetics (e.g., movement/phase durations, time to peak force/power, concentric rate of force development, maximal velocity, eccentric peak power), constraining analyses to performance outcomes (e.g., jump height). Future research should include force-platform measures to elucidate the mechanical determinants of improvement.

## 5 Conclusion

This study indicates that both sand-based PT and hard-surface PT over 8 weeks significantly enhance explosive power, dynamic balance, and agility performance, with no significant differences between groups. Nevertheless, effect sizes reveal a small-to-moderate advantage trend for sand-based PT in explosive power and balance, offering a practical reference value. Therefore, coaches may consider incorporating sand-based PT drills during preparatory or rehabilitation phases to enhance neuromuscular control and reduce impact load while maintaining improvements in balance and explosive strength.

## Data Availability

The raw data supporting the conclusions of this article will be made available by the authors, without undue reservation.
