# Supplementary material for: The effects of eight weeks of sand-based plyometric training on lower-extremity explosive strength, balance, and agility in male collegiate badminton players
Source: Front Physiol. 2025 Nov 26;16:1708619. doi: 10.3389/fphys.2025.1708619 (PMC12689396; doi:10.3389/fphys.2025.1708619)
Supplement: Supplementary file 1 [file DataSheet1.docx]

The Effects of Eight Weeks of Sand-based Plyometric Training on Lower-Extremity Explosive Strength, Balance, and Agility in Male Collegiate Badminton Players

**Nijiao Deng^1, †^, Xin Zheng^1, †^, Hairong Wang^1^, Ziren Zhao^1^, Xu Xia^1^, Hangshuo Zhang^1^,** **Kaixiang Zhou^1^, Bin Zhang^2, *^**

^1^ College of Physical Education and Health Science, Chongqing Normal University, Chongqing, China;

^2^ College of Physical Education and Health Sciences, Zhejiang Normal University, Jinhua, China;

*** Correspondence:** Bin Zhang, PhD, College of Physical Education and Health Sciences, Zhejiang Normal University, Jinhua, China; e-mail address: [zhangbin666@zjnu.edu.cn](mailto:zhangbin666@zjnu.edu.cn)

^†^ **Equal contribution and first authorship:** These authors contributed equally to this work and share first authorship

**Table 1** Plyometric training program.

| **Phase I: Adaptation phase** | **Week 1** | **Week 2** | |
| --- | --- | --- | --- |
| Single-leg vertical hurdle jumps | 8 × 3 | 10 × 3 | |
| Double-leg vertical hurdle jumps | 8 × 3 | 10 × 3 | |
| Single-leg lateral hurdle jumps | 8 × 3 | 10 × 3 | |
| Double-leg lateral hurdle jumps | 8 × 3 | 10 × 3 | |
| Single-leg zig-zag hurdle jumps | 8 × 3 | 10 × 3 | |
| Double-leg zig-zag hurdle jumps | 8 × 3 | 10 × 3 | |
| **Phase II:intensive enhancement phase** | **Week 3** | **Week 4** | **Week 5** |
| Box jumps | 8 × 3 | 10 × 3 | 12 × 3 |
| Depth jumps | 8 × 3 | 10 × 3 | 12 × 3 |
| Lateral push-off box jumps | 8 × 3 | 10 × 3 | 12 × 3 |
| Depth jump+ double-leg hurdle jumps | 10 × 3 | 12 × 3 | 15 × 3 |
| Depth jump + single-leg hurdle jumps | 10 × 3 | 12 × 3 | 15 × 3 |
| **Phase III: maintenance phase** | **Week 6** | **Week 7** | **Week 8** |
| Depth jumps to box | 8 × 3 | 10 × 3 | 12 × 3 |
| Single-leg tuck jumps | 8 × 3 | 10 × 3 | 12 × 3 |
| Half-squat double-leg box jumps | 8 × 3 | 10 × 3 | 12 × 3 |
| Alternating split squat jumps (cyclical) | 10 × 3 | 12 × 3 | 15 × 3 |
| Scissor split squat jumps | 10 × 3 | 12 × 3 | 15 × 3 |

**Table 2** **The effects of sand-based plyometric training on lower-extremity explosive strength, balance, and agility.**

| **Variable** | **Group** | **Pre** | **Post** | **Δ% （Mean）** | **P** | | |
| --- | --- | --- | --- | --- | --- | --- | --- |
|  |  |  |  |  | **Time factor** | **Group factor** | **Interaction** |
| **Explosive strength** | **SJ** | | | | | | |
|  | Sand-based PT | 42.12±8.90 | 47.22±8.44 | ↑12.13% | <0.001 | 0.29 | 0.28 |
|  | Hard-surface PT | 39.36±8.00 | 43.36±4.04 | ↑10.17% |  |  |  |
|  | **CMJ** | | | | | | |
|  | Sand-based PT | 47.74±9.56 | 52.86±9.30 | ↑10.73% | <0.001 | 0.18 | 0.053 |
|  | Hard-surface PT | 44.42±6.67 | 47.67±6.78 | ↑7.31% |  |  |  |
|  | **DJ** | | | | | | |
|  | Sand-based PT | 45.17±9.37 | 50.68±9.36 | ↑12.20% | <0.001 | 0.3 | 0.18 |
|  | Hard-surface PT | 42.88±6.34 | 46.64±6.38 | ↑8.78% |  |  |  |
|  | **LSJ** | | | | | | |
|  | Sand-based PT | 255.71±25.79 | 265.07±27.73 | ↑3.66% | <0.001 | 0.41 | 0.16 |
|  | Hard-surface PT | 248.71±27.19 | 254.86±27.84 | ↑2.47% |  |  |  |
|  | | | | | | | |
| **Y balance** | **Left leg to the forward** | | | | | | |
|  | Sand-based PT | 64.07±7.90 | 70.96±8.49 | ↑10.75% | <0.001 | 0.59 | 0.41 |
|  | Hard-surface PT | 63.14±7.91 | 68.57±9.36 | ↑8.59% |  |  |  |
|  | **Left leg to left** | | | | | | |
|  | Sand-based PT | 109.86±10.34 | 119.93±7.51 | ↑9.15% | <0.001 | 0.36 | 0.31 |
|  | Hard-surface PT | 107.21±12.44 | 115.29±11.88 | ↑7.53% |  |  |  |
|  | **Left leg to right** | | | | | | |
|  | Sand-based PT | 106.79±10.59 | 116.29±8.54 | ↑8.89% | <0.001 | 0.18 | 0.04 |
|  | Hard-surface PT | 102.43±13.63 | 108.86±12.30 | ↑6.28% |  |  |  |
|  | **Right leg to the forward** | | | | | | |
|  | Sand-based PT | 66.07±9.38 | 71.57±9.13 | ↑8.33% | <0.001 | 0.97 | 0.06 |
|  | Hard-surface PT | 67.14±9.34 | 70.79±9.93 | ↑5.42% |  |  |  |
|  | **Right leg to left** | | | | | | |
|  | Sand-based PT | 108.50±9.48 | 117.50±6.98 | ↑8.29% | <0.001 | 0.2 | 0.15 |
|  | Hard-surface PT | 104.18±13.52 | 111.00±12.94 | ↑6.55% |  |  |  |
|  | **Right leg to right** | | | | | | |
|  | Sand-based PT | 112.57±7.98 | 120.18±7.77 | ↑6.76% | <0.001 | 0.36 | 0.16 |
|  | Hard-surface PT | 110.03±12.72 | 115.68±11.09 | ↑5.12% |  |  |  |
|  | | | | | | | |
| **Agility** | **Hexagon jump test** | | | | | | |
|  | Sand-based PT | 16.49±4.01 | 13.37±2.43 | ↓18.89% | <0.001 | 0.63 | 0.23 |
|  | Hard-surface PT | 16.29±2.02 | 14.43±1.32 | ↓11.39% |  |  |  |
|  | **Side-ways agility test** | | | | | | |
|  | Sand-based PT | 17.97±2.07 | 16.66±1.67 | ↓7.29% | <0.001 | 0.38 | 0.87 |
|  | Hard-surface PT | 18.48±2.02 | 17.26±1.18 | ↓6.60% |  |  |  |
|  | **Four-corner agility test** | | | | | | |
|  | Sand-based PT | 45.26±4.97 | 41.09±5.17 | ↓9.22% | <0.001 | 0.93 | 0.23 |
|  | Hard-surface PT | 44.33±5.16 | 41.66±6.07 | ↓6.01% |  |  |  |

SJ, Squat Jump; CMJ, Countermovement Jump; DJ, Drop Jump; LSJ, Long Standing Jump; Δ%, percentage change;


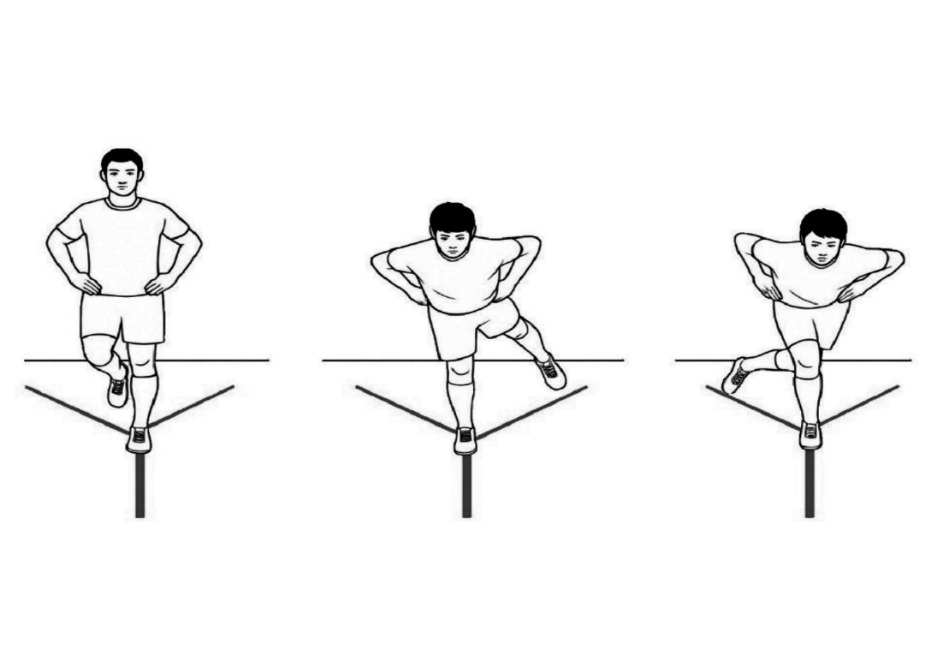


**Figure 1 Y-balance test.**


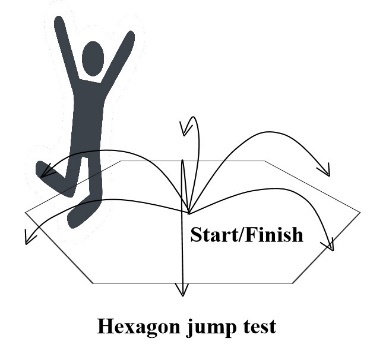


**Figure 2 Hexagon jump test.**


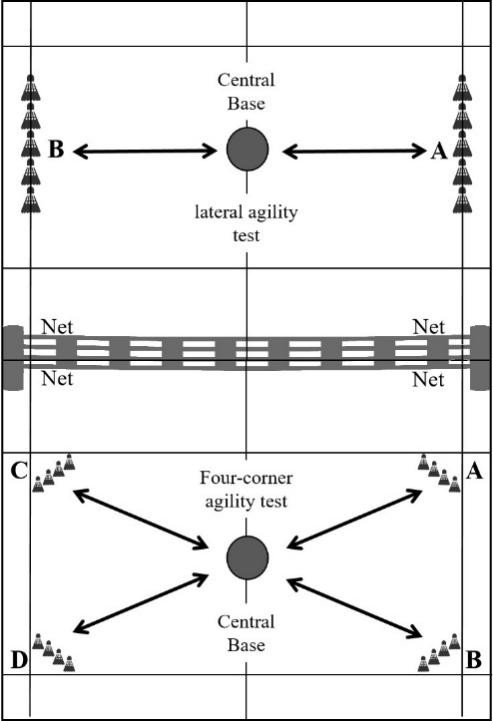


**Figure 3 Badminton-specific agility test.**
